# Fungal Infections and Nail Psoriasis: An Update

**DOI:** 10.3390/jof8020154

**Published:** 2022-02-03

**Authors:** Aikaterini Kyriakou, Sofia-Chrysovalantou Zagalioti, Myrto-Georgia Trakatelli, Christina Fotiadou, Zoe Apalla, Elizabeth Lazaridou, Aikaterini Patsatsi

**Affiliations:** 1 2nd Department of Dermatology and Venereology, Medical School, Aristotle University of Thessaloniki, 56403 Thessaloniki, Greece; mtrakatelli@hotmail.com (M.-G.T.); christinafotiadou@yahoo.com (C.F.); zoimd@yahoo.gr (Z.A.); bethlaz@auth.gr (E.L.); katerinapatsatsi@gmail.com (A.P.); 2 Papageorgiou Hospital, 56403 Thessaloniki, Greece; sofia_zag@yahoo.com

**Keywords:** nail psoriasis, onychomycoses, dermatophytes, non-dermatophyte molds, yeasts

## Abstract

The relationship between psoriasis and onychomycosis is controversial, and the exact nature of this association remains to be clearly elucidated. In healthy nails, the compact nail plate acts as a barrier, preventing any infection. In psoriatic nails, the nail plate involvement, together with abnormalities in the blood capillaries, may lead to decreased natural defenses against microorganisms. Moreover, onycholysis (detachment of the nail plate) induces a humid environment that may favor fungal proliferation. Treatment with immunosuppressive drugs may additionally enhance onychomycosis. In this comprehensive review, we present data regarding the incidence and pathogenic action of dermatophytes and other fungi in the development of fungal infection in psoriatic nails.

## 1. Introduction

The relationship between psoriasis and onychomycosis is controversial, and the exact nature of this association remains to be clearly elucidated. In this comprehensive review, we present data regarding the incidence and pathogenic action of dermatophytes and other fungi in the development of fungal infection in psoriatic nails. We searched the English literature in PubMed using the search terms “onychomycosis” OR “dermatophytic infections” AND “nail psoriasis” OR “psoriatic nails”. There is limited published evidence, mostly from reviews and small cross-sectional and observational studies. There are clinical implications related to this association, including delayed diagnoses of nail psoriasis, unnecessary treatment for onychomycoses, or, on the other hand, undiagnosed fungal infections that may coexist and lead to total onychodystrophy without treatment.

## 2. Nail Psoriasis and Onychomycosis

Psoriasis is a chronic, relapsing, immune-mediated disease, affecting 2–3% of the world’s population [1]. Although skin manifestations are the most characteristic findings of psoriasis, nail involvement is another predominant feature of the disease. The reported prevalence of nail involvement in patients with psoriasis varies between 15% and 79%, whereas the lifetime incidence of nail psoriasis is estimated to reach 80–90% [2,3]. The most common nail alterations of psoriasis are pitting, trachyonychia, leukonychia, red spots in the lunula, subungual hyperkeratosis, onycholysis, splinter hemorrhages and salmon patches [4]. 

Onychomycosis is an infection of the nail caused by dermatophytes, yeasts, and non-dermatophytes molds (NDMs) [5]. It is the most common nail disease and accounts for almost 50% of all nail abnormalities [6]. There are documented risk factors known to raise susceptibility to onychomycosis, such as ageing, gender, genetics, smoking, occlusive footwear, selected professional and hobbistic group activities (swimming pools, saunas, tennis, running, walking barefoot, athletes, coaches, clean workers, housewives) and nail trauma [7]. The most frequent clinical characteristics of onychomycosis are subungual hyperkeratosis, onycholysis and color changes. Onychomycosis can, therefore, resemble nail psoriasis, and sometimes their discrimination is quite difficult on a clinical basis [8,9]. Moreover, coexistence of these two entities may also occur because both are very common in the general population [8,9]. Therefore, physicians are encouraged to use diagnostic techniques, including direct microscopy, culture, and polymerase chain reactions, to verify or rule out onychomycosis in psoriatic patients with nail abnormalities [10]. 

## 3. Morphological Changes in Psoriatic Nails Are Predisposing Factors for Onychomycosis, and Vice Versa

The relationship between psoriasis and onychomycosis is still controversial in the literature, and the exact nature of the association between these disorders remains unclear because they share many common clinical features [11]. In healthy nails, the compact nail plate acts as a barrier preventing fungal infection. In psoriatic nails, except for nail disorders, abnormalities in the blood capillaries are noted, facts that may lead to decreased natural defenses against microorganisms [12]. Moreover, onycholysis (detachment of the nail plate) induces a humid environment that may favor fungal proliferation [10]. Additionally, immunosuppressive drugs may influence susceptibility to developing onychomycosis, especially methotrexate, cyclosporin, infliximab and adalimumab [13,14]. It is reported that they may alter the immune status of patients, predisposing them to infections. Methotrexate significantly slows down the growth of nails, which may also render nails prone to fungal infection [15]. Finally, it has been confirmed that onychomycosis may aggravate nail changes in psoriasis which might occur due to Koebner’s phenomenon [16]. On the other hand, the immune response against microbial skin infections is noticeably strong in psoriasis, and the rapid growth of nails in patients with psoriasis may also play an important role as a prevention against dermatophytes [17]. To date, there is an increased number of serum-like glycoproteins and inhibitor peptides with antifungal activities, whose expression would hinder the development of pathogens in such patients [10]. These contradictory findings lead to a major challenge for more research projects in this field.

## 4. Incidence of Onychomycosis in Psoriatic Nails

The reported incidence of onychomycosis in patients with nail psoriasis varies considerably in the literature and has been found to be equal, lower, or higher compared with that in healthy individuals. The frequency of onychomycosis in this group can range from 4% to 60%, depending on the study [11,18,19].

The prevalence of dermatophyte toenail onychomycosis in psoriatic patients was 10.22%, as cited in the Gupta et al. systematic review paper [20]. It was concluded that the frequency of onychomycosis among inpatients with psoriasis compared with healthy population was significantly higher [20]. A total of 34 studies met the inclusion criteria from a search of 1914 articles. This systematic review included studies from before 2014. The authors estimated the prevalence of culture-confirmed toenail onychomycosis in certain patient populations, including psoriatic patients. Patients with psoriasis had the highest prevalence of non-dermatophyte onychomycosis. Gupta et al. concluded that impaired immunity, reduced peripheral circulation and nail dystrophy seem to be risk factors of toenail onychomycosis in high-risk populations.

In order to draw particular conclusions for all contradictory results of different studies, Klaasen et al. reviewed the available literature to assess the impact of onychomycosis in psoriatic nails [11]: 10 of 723 studies met the inclusion criteria for this systematic review. Six studies were published between 2000 and 2012; two of them in 1990 and the others in 1980. In total, there were 2176 included patients [11]. Unfortunately, a meta-analysis could not be performed due to the significant heterogeneity of the studies. The factors that limited comparability between studies included differences in patient identification, sample size, age group of the population, exact sampling point of the nail that was used for microscopic examination and fungal culture, techniques, and criteria for confirmation of onychomycosis, as well as the lack of clarification of the exact point of onychomycosis (toenail of fingernail). 

More specifically, three studies concluded that the prevalence of onychomycosis was significantly higher in patients with psoriasis compared with healthy individuals. The remaining seven studies reported no significant difference (however, six of them found higher incidence rates in patients with psoriasis). The prevalence of onychomycosis in psoriatic patients varies from 4.6% to 63.1%, whereas in non-psoriatic patients, it ranges from 2.4% to 66.0%. Combining all ten studies, the prevalence of onychomycosis in psoriatic nails was 18%, compared with 9.1% in the control group. Thus, the authors concluded that the frequency of onychomycosis may be higher in patients with psoriasis, but they emphasized the need of further data to confirm this finding.

The positive relationship between psoriatic disease and onychomycosis was also reported by Oliveira Praeiro Alves et al. [13]. They found that the frequency of fungal infections in patients diagnosed with psoriasis and/or psoriatic arthritis was 57.89%. In this study, most patients (32/28) received immunosuppressive therapy (positive relationship, *p* < 0.05). The sample number was small (n = 38); therefore, a broader conclusion, such as if immunosuppressive therapy is a predisposing or a contributing factor of onychomycosis, could not be reached [13]. Zisova et al. commented on the above-mentioned paper indicating a frequency of 62% [16].

Accordingly, Tsentemeidou et al. reported that the prevalence of onychomycosis among patients with nail psoriasis is higher than that among the general population of Greece: 34.78% of patients with psoriasis (total sample size of the study = 23) tested positive for fungal infection [9].

Moreover, a high prevalence of onychomycosis in psoriatic patients was observed by Romaszkiewicz et al. [21]. The presented study showed a 23.53% of onychomycosis in the psoriatic group with clinically abnormal nails. The characteristics of isolated fungi differed significantly between the two groups (psoriatic and non-psoriatic control group). The possible positive relationship between morphological abnormalities in psoriatic nails and fungal invasion was hypothesized [21]. 

## 5. Pathogens in Psoriatic Nails

Numerous studies in the literature have reported the various fungal pathogens that may cause onychomycosis in patients with nail psoriasis and have demonstrated the percentages for the responsible fungi (Table 1). However, evidence has been contradictory and the reason for these rather different results is still not completely understood. 

## 6. Dermatophytes Supported to Be the Most Frequently Involved Fungal Agents

In the study by Zisova et al. among patients who developed onychomycosis of psoriatic nails, dermatophytes were observed in 67% (with an incidence of *T. rubrum* (83%), *T. mentagrophytes* (16%), *T. verrucosum* (1%)), yeasts in 24% (*C. albicans* (85%), *Candida non albicans* (15%)), NDMs in 6% (*Scopulariopsis brevicaulis, Aspergilus niger* and yeast *Rhodotorula* *mucilaginosa*) and mixed infection (*T. rubrum* and *C. albicans*) in 3% of patients [16]. According to the authors, infection of the toenails is frequently due to dermatophytes, whereas yeasts are most common in fingernails.

Kacar et al. showed that the most common type of fungus was dermatophytes in patients with psoriasis and NDMs in a healthy group [19]. Dermatophytes were more common in psoriasis patients than in healthy individuals (*p* = 0.02), whereas the incidence of NDMs was found to be similar between psoriatic and healthy groups. The authors assumed that damage of the keratinocytes due to psoriasis may provide an ideal environment for dermatophytes. *T. rubrum* has been reported as the most common pathogen among dermatophytes in psoriatic patients. The authors concluded that psoriatic nails are a risk factor not for onychomycosis, but specifically for dermatophyte nail infections, and they claimed that the faster growth of psoriatic nails does not protect against dermatophyte infection.

Oliveira Praeiro Alves et al. [13] and Leibovici et al. [22] stated that psoriatic nails are a risk factor for onychomycosis development, especially for dermatophyte nail infection. More specifically, Oliveira Praeiro Alves et al. found that *T. rubrum* was responsible for most cases of onychomycosis, whereas *T. tonsurans* was isolated in only one case of psoriatic patients [13]. In the study by Leibovici et al., dermatophytes were observed in 37.2% of patients with nail psoriasis (*T. rubrum* 35.4%, *T. mentagrophytes* 1.8%), yeasts in 5.2%, and NDMs in 5.2% [22].

Shemer et al. [23] and Jendoubi et al. [24] found that *T. rubrum* was by far the most common dermatophyte cultured from toenails. The only study that observed a similar prevalence of dermatophyte onychomycosis (43.27%) and onychomycosis caused by yeasts (43.55%) in psoriatic patients was that performed by Gallo et al. [25]. This was a 15-year retrospective study in a total of 9281 patients that found no statistically significant difference between the prevalence of onychomycosis in psoriatic patients (49.08%) compared with non-psoriatic patients (51.3%). 

## 7. Yeasts Implicated as Fungal Agents

The incidence of yeast-induced onychomycosis in patients with nail psoriasis is reported to be 19–23% [21]. Several studies have shown that yeasts have a higher probability of developing fungal infection in psoriatic nails. Specifically, in the study published by Rizzo et al. [18], it was reported that in psoriatic patients with onychomycosis, the most common pathogens were *Candida* spp. (69.2%) compared with dermatophytes (30.8%). In contrast, non-psoriatic patients with onychomycosis were more likely to have dermatophyte fungal nail infections. However, the results of this research were not statistically significant (*p* > 0.05).

A 2-year Pakistan study by Tabassum et al. [26] reported that the main fungi pathogen in both psoriatic and non-psoriatic group was *Candida* (27/75) (*Candida parapsilosis* (21/75) as the most common), followed by NDMs—*Aspergillus* spp. (15/75), and last by dermatophytes (5/75). A similar size study by Larsen et al. [27] found that the prevalence of yeasts in psoriatic patients was higher (12.7%; mainly *Candida (C.) krusei*) than the prevalence of dermatophytes (10.1%; mainly *T. rubrum*). This study estimated that there was no statistically significant difference between the prevalence of dermatophytes in both cases and controls. 

Tsentemeidou et al. [9] found that yeast and molds were the main fungal pathogens (37.50% each) isolated by mycological testing. Similar results were reported by Natarajan et al. [28], who concluded that the percentage of fungal culture in psoriatic patients was the same for both yeasts and NDMs (18.75% each).

## 8. NDMs Supported to Be the Most Frequently Involved Fungal Agents

Gupta et al. [20] isolated NDMs (2.49%; 95% CI = 1.74, 3.55) as the highest prevalence of fungi pathogens in individuals with psoriasis. In the groups that were studied, dialysis patients had the highest prevalence of dermatophyte onychomycosis, whereas the elderly had the highest rate of onychomycosis caused by yeasts. Corresponding findings were reported by Romaszkiewicz et al. [21], who concluded that there was a higher probability of developing NDM onychomycosis (*p* = 0.003) in psoriatic patients compared with controls. 

It is obvious that the results in the literature are very heterogeneous. To clarify these data, Klaassen et al. [11] compared the available literature on the prevalence of onychomycosis in patients with nail psoriasis. They also analyzed the predominant pathogens causing onychomycosis, even though this analysis was quite difficult due to the large differences in the criteria and conditions under which the studies were conducted. 

A total of ten studies were analyzed. Five of them concluded that yeasts and/or NDMs were more commonly found in psoriatic nails than in non-psoriatic nails (the difference was statistically significant only in Stander et al.’s study). Three of the studies reported that dermatophytes were the main fungal pathogens between the two comparable populations. 

Overall, dermatophytes were the most commonly found pathogens in onychomycosis in non-psoriatic population and were responsible for up to 90% of toenail fungi infections and up to 50% of fingernail fungi infections (mainly *T. rubrum* and *T. mentagrophytes*, 80–90%). Yeasts and NDMs were rarely isolated as primary pathogens (*Candida* was the most frequent, 1.5–6%). However, due to the large heterogeneity and the qualitative differences between the studies, Klaassen et al. could not reach a conclusion regarding the most common fungi pathogen causing the onychomycosis of psoriatic nails.

## 9. Different Pathogens Reported between Psoriatic Toenails and Fingernails

Various studies have distinguished between different pathogens and reported the incidence rates of agents depending on localization—toenails or fingernails. According to the study by Shemer A. et al., dermatophytes were the most common pathogens found in cultures from toenails, and yeasts from fingernails [23]. Among dermatophyte isolates, *T. rubrum* (60.8%) was the most common fungal toenail infection and *C. tropicalis* (40%) was the most common yeast in fingernail onychomycosis. Moreover, the most frequent causative agents were also dermatophytes for toenails and yeasts for fingernails in another study by Gallo L. et al. [25]. Szepietowski J.C. and Salomon J. also reported a higher frequency of dermatophytes (31.5%) in toenail fungal infections [8]. However, onychomycosis of the hands and feet in patients with psoriasis was caused specifically by NDMs (*Aspergillus niger* for both fingernails and toenails) [28]. Contrasting results were also concluded by Chaowattanapanit S. et al. They evaluated a higher probability of yeasts in fingernails (41.9%) and NDMs in toenails (32.2%) [29]. Therefore, controversies remain in this field of the reported fungal isolation agents among toenail and fingernail onychomycosis.

## 10. The Effect of Psoriasis Treatment on the Incidence of Onychomycosis

Treatment against psoriasis usually involves the use of either topical corticosteroids or systemic immunosuppressive drugs, such as cyclosporine, methotrexate, and biological agents. The application of topical steroids to treat nail psoriasis seems to be a factor favoring the fungal infection, due to their immunosuppressive effect [10]. Cyclosporine also alters the immune status of patients and may predispose psoriatic nails to onychomycosis [14]. It has been reported that methotrexate, except for its immunosuppressive effect, significantly slows down the nail growth, making nails prone to fungal infection [15]. 

In the last decade, the use of biological agents in the management of psoriasis has remarkably increased. It has been speculated that as immunomodulators, these drugs may increase the risk of onychomycosis. Al Mutaifi et al. found that the incident of onychomycosis in psoriatic patients with nail involvement receiving anti-TNF-α was 20.3%, compared with 13.9% of controls [30]. They reported that this risk was significantly higher for infliximab, and they concluded that there is a significant association between onychomycosis and patients with nail psoriasis receiving anti-TNF treatment [30]. Recently, Oliveira Praeiro Alves et al. proved that the uses of systemic treatments against psoriasis and onychomycosis were positively related (*p* < 0.05) [13]. Patients without treatment or on acitretin did not have an increased risk for onychomycosis. On the other hand, patients on methotrexate had a 92.8% positivity rate for onychomycosis (*p* < 0.05). Increased rates of onychomycosis were also reported in patients treated with adalimumab and infliximab. 

This evidence may indicate that immunosuppressive drugs could contribute to the increased risk of onychomycosis in patients with nail psoriasis. However, the literature on the role of immunosuppressive agents as contributing factors for fungal infections is extremely limited, and further studies are needed to draw reliable conclusions.

## 11. Conclusions

Onychomycosis and nail psoriasis are common diseases affecting nails. The clinical manifestations of onychomycosis frequently resemble nail psoriasis and vice versa, although the clinical differentiation of the two entities is sometimes complicated. The relationship between psoriasis and onychomycosis is still controversial in the literature, and the exact nature of the association between these disorders remains unclear. However, a tendency towards higher prevalence numbers of onychomycosis in psoriasis patients has been suggested. Moreover, there is some evidence indicating that the widely used immunosuppressive drugs may contribute to the increased risk of onychomycosis in this group of patients. 

On a clinical basis, we suggest that psoriatic patients with nail lesions which are resistant to treatment or have clinical sings of onychomycosis should be checked through direct microscopy and culture. Sometimes nail psoriasis and onychomycosis co-exist. In such cases, systemic antifungal treatment is indicated, based on the causative fungus, and lasting for 3–4 months. During that time, topical steroids applied on the nail unit should be avoided, because they may aggravate fungal nail infection. 

With this study, we present recent data and highlight the frequency of fungal infections in nail psoriasis and the importance of nail sampling and culture, in order to neither underdiagnose nor to overtreat onychomycoses.

## Figures and Tables

**Table 1:** Characteristics of individual studies included in this review. Prevalence of isolated fungi and isolated fungal species in psoriatic nails.

Study	Group of Fungi, %	Causative Agents
Zisova, L. et al. [16]	Dermatophytes, 67%	*Trichophyton rubrum*
		*Trichophyton mentagrophytes*
		*Trichophyton verrucosum*
	Yeasts, 24%	*Candida albicans*
		*Candida non-albicans*
	NDMs, 6%	*Scopulariopsis brevicaulis*
		*Aspergillus niger*
		*Yeast* *Rhodotorula* *mucilaginosa*
	Mixed infection, 3%	Combination of *Trichophyton rubrum* and *Candida albicans*
Kacar, N. et al. [19]	Dermatophytes, 61.5%	*Trichophyton rubrum*
	Yeasts, 23%	*Saccharomyces cerevisiae*
		*Candida parapsilosis*
		*Candida guilliermondii*
	NDMs, 15.3%	*Alternaria* sp.
Alves, N. et al. [13]	Dermatophytes, 56.3%	*Trichophyton rubrum*
		*Trichophyton tonsurans*
	Yeasts, 43.75%	*Candida parapsilosis*
		*Candida albicans*
Leibovici, V. et al. [22]	Dermatophytes, 37.2%	*Trichophyton rubrum*
		*Trichophyton mentagrophytes*
	Yeasts, 5.2%	*Candida albicans*
		*Candida parapsilosis*
		*Candida* sp.
	NDMS, 5.2%	*Scopulariopsis* *brevicaulis*
Shamer, A. et al. [23]	Dermatophytes, 51.5%	*Trichophyton rubrum*
		*Trichophyton mentagrophytes*
	Yeasts, 21.2%	*Candida albicans*
		*Candida tropicalis*
	NDMs, 27.2%	*Aspergillus fumigatus*
		*Aspergillus niger*
		*Scopulariopsis brevicaulis*
Jendoubi, F. et al. [24]	Dermatophytes	*Trichophyton rubrum*
	Yeasts	*Candida parapsilosis*
		*Candida albicans*
		*Candida non-albicans*
Gallo, L. et al. [25]	Dermatophytes, 43.27%	*Trichophyton mentagrophytes*
		*Trichophyton rubrum*
	Yeasts, 43.55%	*Candida albicans*
		*Candida parapsilosis*
		*Candida krusei*
		*Candida tropicalis*
	NDMs, 13.18%	*Aspergillus fumigatus*
		*Aspergillus niger*
		*Penicillium*
		*Cephalosporium*
		*Cladosporium*
		*Scopulariopsis brevicaulis*
		*Alternaria* sp.
Rizzo, D. et al. [18]	Dermatophytes, 56.9%	
	Yeasts, 43.1%	*Candida* spp.
Tabassum, S. et al. [26]	Dermatophytes, 6.6%	*Trichophyton rubrum*
		*Trichophyton interdigitale*
	Yeasts, 36%	*Candida parapsilosis*
		*Candida albicans*
		*Candida tropicalis*
		*Candida krusei*
	NDMs, 40%	*Aspergillus flavus*
		*Aspergillus niger*
		*Aspergillus terreus*
		*Bipolaris*
		*Fusarium*
		*Alternaria*
		*Scopulariopsis*
		*Scytalidium*
		*Acremonium*
	Mixed infection, 17.3%	
Larsen, G.K. et al. [27]	Dermatophytes, 10.1%	*Trichophyton rubrum*
		*Trichophyton mentagrophytes*
	Yeasts, 12.7%	*Candida albicans*
		*Candida krusei*
		*Trichosporon beigelii*
	NDMs, 4%	
Tsentemeidou, A. et al. [9]	Dermatophytes, 12.5%	*Trichophyton rubrum*
	Yeasts, 37.5%	*Candida albicans*
		*Candida parapsilosis*
	NDMs, 37.5%	*Scopulariopsis brevicaulis*
		*Aspergillus* sp.
Natarajan, V. et al. [28]	Yeasts, 18.75%	*Candida tropicalis*
		*Candida parapsilosis*
		*Candida glabrata*
		*Trichosporon* sp.
	NDMs, 18.75%	*Aspergillus niger*
		*Syncephalastrum* sp.
		*Penicillium* sp.
		*Aspergillus flavus*
Romaszkiewicz, A. et al. [21]	Dermatyophytes, 7%	*Trichophyton rubrum*
		*Trichophyton mentagrophytes*
	Yeasts, 12%	*Candida albicans*
		*Candida glabrata*
		*Candida krusei*
		*Geotrichum candidum*
	NDMs, 5%	*Scopulariopsis brevicaulis*
